# Exercise Rescues Obesogenic-Related Genes in the Female Hypothalamic Arcuate Nucleus: A Potential Role of miR-211 Modulation

**DOI:** 10.3390/ijms25137188

**Published:** 2024-06-29

**Authors:** Kayla Rapps, Asaf Marco, Hilla Pe’er-Nissan, Tatiana Kisliouk, Gabrielle Stemp, Gal Yadid, Aron Weller, Noam Meiri

**Affiliations:** 1 Faculty of Life Sciences, Bar Ilan University, Ramat Gan 5290002, Israel; kaylagasner@gmail.com (K.R.); peer.hilla@gmail.com (H.P.-N.); yadidg@mail.biu.ac.il (G.Y.); 2 Institute of Animal Science, Agricultural Research Organization, The Volcani Center, Rishon LeZion 7528809, Israel; tatiana.kisliouk@mail.huji.ac.il; 3 Gonda Multidisciplinary Brain Research Center, Bar Ilan University, Ramat Gan 5290002, Israel; gaby.stemp@gmail.com (G.S.); Aron.weller@biu.ac.il (A.W.); 4 Neuro-Epigenetics Laboratory, Faculty of Agriculture, Food and Environment, The Hebrew University of Jerusalem, Rehovot 7610001, Israel; asaf.marco@mail.huji.ac.il; 5 Department of Psychology, Bar Ilan University, Ramat Gan 5290002, Israel

**Keywords:** obesity, gene expression, hypothalamus, voluntary exercise, diet-induced obesity, epigenetics, micro-RNA

## Abstract

Obesity is a major public health concern that is associated with negative health outcomes. Exercise and dietary restriction are commonly recommended to prevent or combat obesity. This study investigates how voluntary exercise mitigates abnormal gene expression in the hypothalamic arcuate nucleus (ARC) of diet-induced obese (DIO) rats. Using a transcriptomic approach, novel genes in the ARC affected by voluntary wheel running were assessed alongside physiology, pharmacology, and bioinformatics analysis to evaluate the role of miR-211 in reversing obesity. Exercise curbed weight gain and fat mass, and restored ARC gene expression. High-fat diet (HFD) consumption can dysregulate satiety/hunger mechanisms in the ARC. Transcriptional clusters revealed that running altered gene expression patterns, including inflammation and cellular structure genes. To uncover regulatory mechanisms governing gene expression in DIO attenuation, we explored miR-211, which is implicated in systemic inflammation. Exercise ameliorated DIO overexpression of *miR-211*, demonstrating its pivotal role in regulating inflammation in the ARC. Further, in vivo central administration of miR-211-mimic affected the expression of immunity and cell cycle-related genes. By cross-referencing exercise-affected and miR-211-regulated genes, potential candidates for obesity reduction through exercise were identified. This research suggests that exercise may rescue obesity through gene expression changes mediated partially through miR-211.

## 1. Introduction

The benefits of living a healthy lifestyle—requiring regular exercise and a balanced diet—are widely acknowledged. However, maintaining such a lifestyle can be challenging for many, especially for those who are overweight. This prompts the question: Is there merit in engaging in intermittent voluntary exercise without making further lifestyle adjustments? Research conducted by ourselves and others has shown, in animal models, that obesity involves maladaptation of the satiety/hunger center in the hypothalamic arcuate nucleus (ARC) and it may entail low-level inflammation of this nucleus. Despite evidence demonstrating the benefits of exercise, the molecular pathways involved are only beginning to be understood. In this study, we delve into this question at the molecular and epigenetic levels by identifying the array of genes rescued by exercise in an obesogenic context in the ARC, with a particular focus on immune-related genes and offering the potential involvement of a specific microRNA. Diet-induced obesity (DIO) and consumption of high-fat diet (HFD) induces dysfunction in peripheral metabolically-active organs and the hypothalamus, resulting in energy homeostasis dysregulation [1]. The hypothalamic arcuate nucleus (ARC) is responsible for numerous neuroendocrine functions and integration of environmental and peripheral feedback to maintain energy homeostasis [2]. In a state of obesity, molecular changes in the ARC disturb normal metabolic feedback, perpetuating weight gain and overconsumption.

Diverse mechanisms orchestrate the perturbation of hypothalamic function in the context of obesity. In the ARC, extended HFD exposure reduces synaptic transmission [3], increases programmed cell death [4], and decreases neuronal turnover [4]. Chronic low-grade inflammation in the ARC is correlated with obesity, and both peripheral organs and the hypothalamus show signs of inflammation through weight gain. In the ARC, HFD promotes inflammatory response through chemokine/cytokine release and astrocyte and macrophage recruitment [5].

Sedentary lifestyle is a significant factor leading to obesity and metabolic syndrome (MeS). It has been established that exercise, even in the absence of diet changes elicits beneficial health outcomes [6]. In overweight rats, regular bouts of activity lead to improvements in cognitive function [7], insulin sensitivity [8], glucose metabolism [8], lipid profile [9], and overall reduction of systemic inflammation [10]. Notably, it has been shown that voluntary exercise is effective in promoting molecular and epigenetic regulation within the hypothalamus, leading to improvements in physiological traits such as stress and obesity [11,12,13]. Furthermore, as low grade inflammation has been correlated with hypothalamic dysfunction and obesity, current research has demonstrated that exercise effectively diminishes neuroinflammation [14] through the induction of anti-inflammatory markers, such as interlukin-6 (IL-6) and interlukin-10 (IL-10), coupled with a reduction in local cellular stress response [15]. 

We [16,17,18] and others [19,20] have demonstrated that transcriptional modulation in the ARC contributes to obesity and MeS, and that this effect can be reversed through weight loss. Although improvement of physiological symptoms associated with MeS have been established in obese-exercise models, our focus was on conducting a comprehensive assessment of transcriptional programs. Specifically, we were interested in exploring the role of microRNA (miR) modulation in reversal of obesogenic-related gene expression in the hypothalamus. 

miR-211 is a conserved regulator of gene expression throughout peripheral organs and the brain. It has complex regulatory roles in cancers [21] and has recently been identified as a biomarker for metastasis of breast cancer [22]. Moreover, miR-211 plays a pivotal role regulating anti-inflammatory response by inhibiting lipopolysaccharide (LPS)-induced inflammation [23]. Notably, miR-211 has been established as a regulator of neuroinflammation in the context of neurovascular episodes and neurodegenerative diseases [24]. Critically, exercise before a stroke/ischemic episode protects against progression of ischemia and increases survival by upregulating miR-211 in the brain [25]. We used a combined approach of physiology, pharmacology, and transcriptomic and bioinformatics analysis to determine the role of miR-211 in the control of energy balance, and importantly, the reversal of obesity-induced hypothalamic inflammation through voluntary exercise. 

## 2. Results

### 2.1. Voluntary Exercise Reduces Weight Gain and Body Fat in Diet-Induced Obese Rats

**Figure 1:**
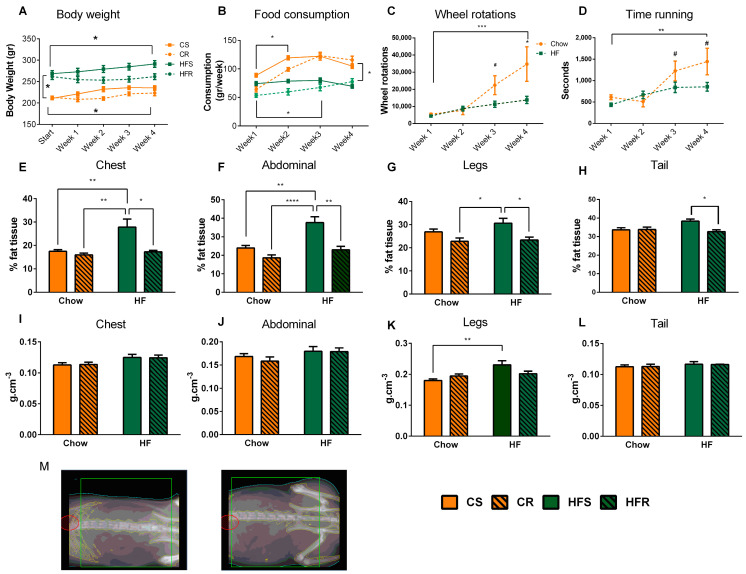
Physiological effects of voluntary exercise on diet-induced obese female Wistar rats. (**A**) Body weight at start point of voluntary activity (PND 90) in specialized exercise chambers, and weekly through experiment. Rats were sacrificed at the end of week 4 (PND 120). Two-way repeated ANOVA revealed a significant main effect of time (F_(4,172)_ =35, *p* < 0.0001) and significant main effect of diet (F_(3,43)_ =24, *p* < 0.0001), and a significant interaction of time and diet on body weight (F_(12,172)_ = 7.8, *p* < 0.0001). (**B**) Weekly food intake. Two-way repeated ANOVA reveled a significant main effect of time (F_(3,74)_ = 16.22, *p* < 0.0001), diet (F_(3,74)_ = 1.251, *p* = 0.27) and non-significant interaction F_(3,74)_ = 0.81, *p* = 0.48 on intake. (**C**) Number of wheel rotations per week. Two-way repeated ANOVA revealed a significant effect of time (F_(3,70)_ = 7.626, *p* < 0.0001) and a significant effect of diet (F_(1,70)_ =6.039, *p* < 0.05, but no significant interaction (F_(3,70)_ = 2.34, *p* = 0.072) with weekly wheel rotations. (**D**) Time (s) spent running on wheels per week. Two-way ANOVA revealed a significant effect of time (F_(3,72)_ = 7.23, *p* < 0.001) and a significant main effect of diet (F_(3,72)_ = 4.27, *p* < 0.05) but no significant interaction (F_(3,72)_ = 1.57, *p* = 0.20). (**E**–**H**) Percentage of body fat of the (**E**) chest (ANOVA: main effect of diet: F_(1,16)_ = 6.7, *p* < 0.05; main effect of activity: F(_1,16)_ = 7.2, *p* < 0.05; interaction of diet x activity: F_(1,16)_ = 4.0, *p* = 0.06); (**F**) abdomen (ANOVA main effect of diet: F_(1,16_ = 14, *p* < 0.001, main effect of activity: F_(1,16)_ = 17, *p* < 0.001, interaction of diet x activity: F_(1,16)_ = 3.8, *p* = 0.07); (**G**) legs (ANOVA main effect of diet: F_(1,17)_ = 1.7, *p* < 0.05, main effect of activity: F_(1,17)_ = 12, *p* < 0.01, interaction of diet x activity: F_(1,17)_ =0.95, *p* = 0.34); (**H**) tail (ANOVA main effect of diet: F_(1,17)_ = 2.0, *p* =0.18, main effect of activity: F_(1,17)_ = 4.7, *p* < 0.05, interaction od diet x activity: F_(1,17)_ =5.4, *p* <0.05). (**I**–**L**) Bone mass density (BMD) of (**I**) chest, (**J**) abdomen, and (**L**) tail; No difference between treatments; (**K**) legs (ANOVA main effect of diet: F_(1,17)_ = 9.5, *p* < 0.01, main effect of activity: F_(1,17)_ =0.52, *p* < 0.48, interaction diet x activity: F_(1,17)_ = 5.2, *p* < 0.05). (**M**) Captured images from DEXA of abdominal region of HFS rat and HFR rat. Data are presented as means ± SEM. Group differences were revealed using Tukey’s post hoc analysis, and significance is indicated as * 0.01 < *p* < 0.05, ** 0.001 < *p* < 0.01, *** *p* < 0.001, **** *p* < 0.0001, # *p* < 0.1 (trend). n = 10–12 biological replicates per group for behavioral analysis (feeding, body weight, activity); n = 5–6 biological replicates per group for DEXA analysis.

Exercise plays a pivotal role in reversing the metabolic symptoms linked to obesity. Thus, we used a model of voluntary running (after DIO was established) to assess both the physiological benefits and the corresponding transcriptional patterns in the arcuate nucleus (ARC). Rats were assigned to one of the four treatment groups with care to equalize body weight at weaning between HF and control diet groups and before exercise onset between sedentary and exercising groups: Chow-Sedentary (CS), Chow running (CR), High-fat Sedentary (HFS), and High-fat Running (HFR). First, we evaluated the effects of voluntary running on body weight, food consumption, and body composition. Rats that received HFD from the weaning period onward exhibited a significantly higher weight at postnatal day 90 (adulthood) compared to the chow-fed rats (*p* < 0.001). This weight difference persisted consistently throughout the four-week exercise phase, irrespective of activity levels (Figure 1A). At each time point from week 2 and onward, the running groups weighed significantly less than the sedentary groups, within each respective diet group (CR less than CS, *p* < 0.001; HFR less than HFS, *p* < 0.01). 

Both HFD-fed and chow-fed runners increased their food intake throughout the experiment (*p* < 0.05; Figure 1B), while the sedentary groups consumed similar amounts of food. It should be noted that when converting the consumption into calories, the HFD-fed groups initially consumed significantly more, but this difference diminished at the end of the 4 weeks’ experiment. 

Both groups increased activity over time, shown by increased rotations (*p* < 0.001; Figure 1C) and time spent on the wheel (*p* < 0.05; Figure 1D). Both running groups covered similar distances (Figure 1C) during weeks 1 and 2, and the CR group ran a longer distance in weeks 3 and 4 compared to HFR (*p* < 0.05). 

Body composition analysis assessed by DEXA revealed that HFS had increased fat mass compared to CS in the chest (*p* < 0.001) and abdominal regions (*p* < 0.001), but no differences in fat mass were found in the legs or tail. Further, exercise reduced fat tissue compared to HFS in all four anatomical regions sampled (chest: *p* < 0.05; abdomen: *p* < 0.001; legs: *p* < 0.05; tail: *p* < 0.05; Figure 1E–H). The legs of obese sedentary animals (CS) presented a higher bone mineral density (BMD; g/cm^3^) compared to lean sedentary animals (*p* < 0.01) (Figure 1K) with no other region or group affected (Figure 1I,J,L).

### 2.2. Voluntary Physical Activity, on the Background of HFD Affects the Expression of Functional Gene Families in the ARC

Because of the physiological benefits of exercise, we were interested in exploring whether exercise of HFD-fed rats could modify the dysregulation of gene expression in the ARC. To this end an RNA-Seq was performed on the hypothalamic ARC nucleus of the four treatment groups i.e., CS, CR, HFS, and HFR. Principal component analysis (PCA) clearly illustrated a distinct clustering of the CS group, showcasing marked differences from the HFS group (accounting for 43% variation on the PC1, Supplementary Figure S1). Furthermore, the HFR group cluster closely to the CS group, suggesting a potential rescue effect. As each rat exhibited individual performance when exposed toIn thtfff voluntary running wheels, as expected, there was a higher expression variance within the HFR group compared to the sedentary groups (Figure S1). However, in the HFR group, only one subject deviated from the main cluster and thus it was not excluded from the analysis. 

**Figure 2:**
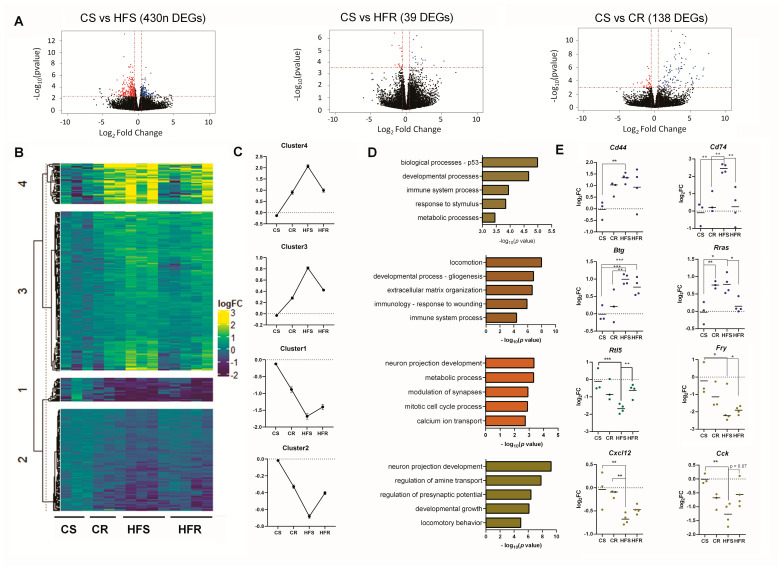
**Transcriptional landscape of differentially expressed genes (DEGs) in the arcuate nucleus.** (**A**) Pairwise analysis of DEGs from the ARC of CS and HFS; CS and HFR and CS and CR are shown in a volcano plot (FDR < 0.1; log_2_FC > 0.5, n = 3 (CS, CR) or 4 (HFS, HFR) biologically independent samples. (**B**) Heat map and (**C**) line graphs show the average transcriptional changes (log_2_FC), compared to the CS group. Gene-sets were clustered by K-means to clusters 1 through 4. Mean of 3 (CS and CR) or 4 (HFS and HFR) biologically independent samples. (**D**) Gene ontology (GO) analysis (ToppFun) for the DEGs that were identified in each cluster. (**E**) Representative DEFs from each cluster. Log_2_FC from the CS is presented in 3 (CS and CR) or 4 (HFS and HFR) biological replicates. Each dot represents a single animal. Data are presented with a line mean. Significant effect between groups is indicated by * 0.01 < *p* < 0.05, ** 0.001 < *p* < 0.01, *** *p* < 0.0001, using ANOVA test with Tukey’ post-hoc test for multiple comparisons.

The first objective was to quantify differentially expressed genes (DEGs) of the three groups (CR, HFS, and HFR), relative to the control CS group. These pairwise comparisons uncovered 265 downregulated genes and 165 upregulated genes in CS vs. HFS; 19 downregulated genes and 20 upregulated genes in CS vs. HFR, and 35 downregulated genes and 93 upregulated genes in CS vs. CR (FDR < 0.1, Figure 2A). 

This analysis generated four clusters each with a distinctly unique pattern of gene expression (Figure 2B,C). Across all clusters, we identified a consistent pattern in gene expression changes, characterized by a significant increase or decrease in the CR group which was intensified in the HFS group, followed by a corrective expression (returning towards the baseline of control chow-fed CS) in the HFR group. Pairwise analysis of the average gene expression within each cluster revealed significant differences between HFS and HFR groups, across all comparisons (Figure 2C, *p* < 0.0001 in all clusters). Additionally, significant differences were identified between CS and both CR and HFS groups in all clusters (*p* < 0.0001 for CS vs. CR and CS vs. HFS). It should be noted that the voluntary exercise rescue effect was partial as the pairwise comparison between CS and HFR also showed a significant difference between the average gene expression within each cluster (Figure 2C; in all clusters, *p* < 0.0001). Together, the data support the notion that voluntary running exerts a partial rescue effect in DIO rats, mitigating the gene expression changes induced by high-fat sedentary (HFS) conditions and reverting them towards the baseline observed in the control chow-fed (CS) group. The effects on gene expression of chow-fed running (CR) were opposite to that of HFR. It should be noted that due to the voluntary nature of the running parameter, there is variation between the rats, which is likely reflected in the gene expression response (the list of genes in each cluster is depicted in Table S1).

We used GO analysis to understand the biological function of each of the gene clusters (Figure 2D). Representative biologically-relevant genes from each cluster are depicted in Figure 2E. In cluster 1, *Rescue down*, i.e., HFS is downregulated and maintains low expression with some rescue effect in HFR. The transcriptional signature of this cluster was enriched for genes involved in regulation of ‘calcium ion transport’ and ‘modulation of synapses’. Within this cluster, we identified *Rtl5*, recognized as a microglial gene with pivotal roles in the forefront of the innate brain immune response [26]. In the second cluster (cluster 2), *Rescue down*, genes that were initially downregulated in HFS conditions were observed to be rescued upward through running. Interestingly, genes enriched in this cluster were involved in ‘locomotion behavior’, ‘developmental growth’, and ‘presynaptic potentials’. A notable gene within this cluster is *Cck*., Cholecystokinin (CCK), has been demonstrated to regulate satiety, with several sites of action identified in the hypothalamus [27]. Another gene within this cluster, *Cxcl12*, is correlated with learning and has been observed to be upregulated in exercise models [28]. The largest cluster (*Rescue up*, 210 genes, cluster 3), was enriched for genes regulating ‘immune system processes’, ‘wound healing’, and ‘organization of extracellular structures’. The expression in the HFR is slightly downregulated yet significant compared to HFS. Within this cluster, we pinpointed *Btg2* and *Rras*, both of which were significantly upregulated in the HFS group, with a noteworthy modest rebound observed in the HFR group. *Rras*, which is rescued in this cluster, may serve as a strong link between the innate immunological system and obesity [29]. Cluster 4, *Rescue up*, featured genes that were markedly upregulated in the HFS group and exhibited a downregulation (trend towards the CS) in the HFR condition. This cluster was characterized by genes involved in the immune response, as seen by enrichment of GO terms ‘T-cell differentiation’, ‘wound healing’, ‘glial cell development’, and ‘cytokine response’. Genes in this cluster include *Cd44*, *Cd74*, *Mt2a*, *Cxcl16*, and *HSPB*. This cluster further includes *Pomc*, a well-established anorexigenic factor, which, as demonstrated by our research and others, exhibits an altered expression pattern in obesity [16,17,18,19,20]. 

Collectively, as observed in previous studies, our results affirm the beneficial effects of running on physiology and gene expression. Furthermore, our findings indicate that even in the context of obesity, voluntary running can result in a partial rescue of hypothalamic gene clusters. Because of the vast transcriptome changes in the model, we next aimed to understand the regulation of these DEGs via microRNA modulation. 

### 2.3. miR-211 Modulates Feeding Behavior

While many miRs have been associated with energy balance and obesity development, we sought to uncover a novel miR that plays a regulatory role in modulating gene expression within our experimental system. In our initial in silico analysis of DEGs, we identified a considerable number of predicted miRs potentially involved in the regulation of these genes. Notably, miR-211 stood out among them, as it has been reported in previous studies to be highly active in the brain [30] and play a crucial role in controlling processes such as inflammation suppression reduction and immune regulation [24,25]. Thus, we hypothesized that miR-211 might play a role in regulating genes within the ARC under the conditions of DIO and exercise, contributing to the maintenance of a low inflammatory rate. 

**Figure 3:**
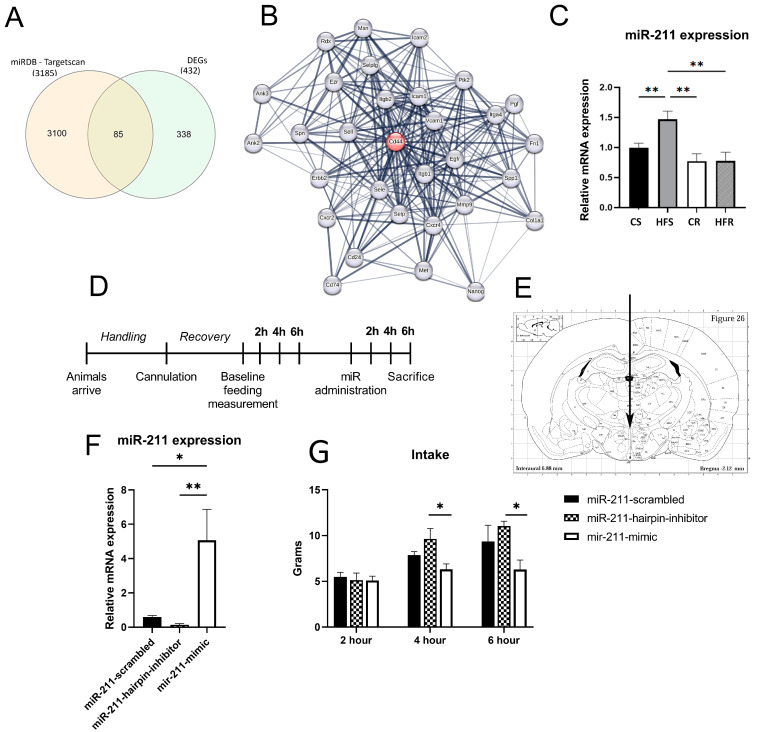
**Effects of central miR-211 administration on feeding in naïve rats.** (**A**) Comparison between the differentially expressed genes and the in silico predicted mRNA targets of miR-211. (**B**) Predicted network analysis using STRING, version 12.0, of the connections of *Cd44* influence 26 of the identified DEGs in Figure 2. (**C**) Transcriptional expression of *miR-211* in the ARC of CS, CR, HFS, and HFR samples measured using RT-qPCR. Relative gene expression if the CS group was set to 1. (**D**) Schematic outline for in vivo miR-injection study. In brief, naïve rats were cannulated with the aid of a stereotactic instrument followed by a recovery period. Food was removed overnight before baseline food intake monitoring to ensure a uniform hunger level between rats at the start of feeding monitoring. Feeding was monitored at three time points for a total of 6 h. One week later, rats were injected with 1 μg of MiRIDIAN hsa-miR-211 mimic, MiRIDIAN hsa-miR-211-hairpin inhibitor, or MiRIDIAN mimic-negative control over a 5 min period and then returned to test cages for feeding monitoring at three time points for a total of 6 h, after which the experiment was terminated. (**E**) Illustration of cannula placement based on stereotactic coordinates AP: −2.12 mm; L: 0 mm; DV: 7.5 mm (from Bregma), shown on the brain atlas. (**F**) After the rats were sacrificed; the ARC was extracted. Relative expression of *miR-211* from extracted ARC using primers specific for *miRNA-211* quantification. (**G**) Feeding profile of rats 2, 4, and 6 h after compound administration; food consumed was briefly removed from each cage at the designated time and weighed manually. Statistical significance at each time point was assessed ANOVA (2 h: F_(2,23)_= 0.99, *p* = 0.38; 4 h: F_(2,23)_= 3.75, *p* < 0.05; 6 h: F_(2,23)_ = 4.25, *p* < 0.05). Statistical significance of gene expression was assessed with ANOVA: F_(2,22)_ = 7.29, *p* < 0.01. Data are presented as mean ± SEM. Significant effect between groups is indicated by * 0.01 < *p* < 0.05, ** 0.001 < *p* < 0.01. Statistical significance for feeding behavior and expression was performed using ANOVA test with Tukey’s test for multiple comparisons.

To ascertain the relevance of assuming a role for miR-211 in the rescue of obesogenic traits through exercise, we compared our list of DEGs (Figure 2) with the potential targets of miR-211, as identified by miRDB and Targetscan (Figure 3A). Among the 432 DEGs, 85 were identified as potential targets of miR-211. Notably, these potential targets included immunologically related genes such as *Cd44*. We further analyzed our identified DEGs, focusing on pathways affected by miR-211. This analysis demonstrated that a substantial number of genes in our list of altered genes could be indirectly influenced by changes in the expression of *miR-211*. To further investigate this hypothesis, we conducted a comprehensive interaction analysis on the DEGs (STRING, version 12.0). We specifically examined various levels of gene and protein interactions, including gene fusion events, phylogenetic co-occurrence, homology, co-expression, experimentally validated interactions, and curated database annotations. Our analysis demonstrated a significant enrichment of protein or gene interactions (*p*-value < 1 × 10^16^) within the DEGs. Specifically, we observed 873 interactions among the DEGs, markedly higher than the 536 interactions expected for a random set of proteins/genes of similar size and degree of distribution (Supplementary Figure S2 and Supplementary Table S2). This enrichment suggests a biological interconnection among these DEGs as a group. Importantly, we identified that 48 of the 85 overlapping genes (i.e., predicated—DEGs match) exhibited significant interactions (combined score > 0.4) with 320 other DEGs. Among the most notable interactions, *Cd44* was predicted to influence 26 DEGs (Figure 3B), while *Gad2*, *Cxcl12*, and *Aldh1a1* also emerged as key interacting genes. These findings underline the intricate network of gene interactions and the potential direct and indirect regulatory effects of miR-211 on the expression of DEGs. 

To verify the involvement of miR-211 in obesity and its potential rescue by exercise, we evaluated its expression levels in the Arc in the four treatment groups (i.e., CS, CR, HFS, and HFR). We found that *miR-211* levels were significantly upregulated in the HFS group compared to the CS group (*p* < 0.05) and significantly downregulated in the HFR condition (*p* < 0.01) [F(_3,42_) = 7, *p* < 0.01] (Figure 3C).

MiR-211 is known as an anti-inflammatory agent [25,31], yet its specific role within the hypothalamus and its effects on energy regulation remain unknown. Therefore, to probe the post-transcriptional mechanism which may regulate hypothalamic dysregulation, we designed an experiment to explore the possibility of feeding effects induced by miR-211 administration. MiRIDIAN-hsa-miR-211 mimic, MiRIDIAN-hsa-miR-211-hairpin inhibitor, or MiRIDIAN-mimic-negative control was delivered directly to the ARC via the 3rd ventricle (Figure 3D,E). After miR-administration, rats were returned to testing caging for feeding measurement with intake measured 2, 4, and 6 h after administration (Figure 3D). 

The effectiveness of miR-211 transfection was evaluated by measuring in all groups, *miR-211* expression in the ARC. The five-fold expression of *miR-211* in the miR-211-mimic group compared to the miR-211-negative control group (*p* < 0.05) and the miR-211-hairpin-inhibitor group (*p* < 0.01) confirmed proper transfection (Figure 3F). Two hours post-administration there were no significant differences in food intake (*p* = 0.89); however, at 4 h (*p* < 0.05) and 6 h (*p <* 0.05) post-administration, a notable reduction in feeding was evident in the miR-211-mimic group compared to the miR-211-hairpin inhibitor (control) group (Figure 3G). 

### 2.4. miR-211 Is Involved in Inflammation and Cell Cycle Regulation

**Figure 4:**
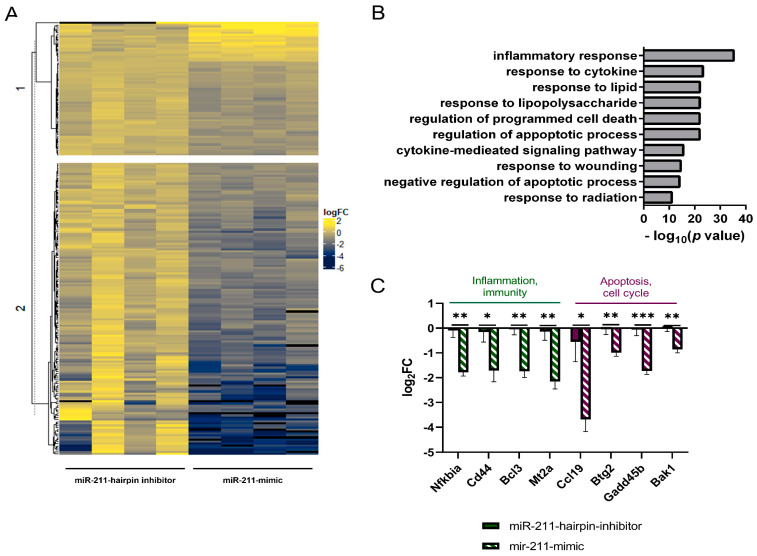
**miR-211 inhibits gene expression related to inflammation and cell cycle in the ARC.** (**A**) Heat map depicting the transcriptional changes of differentially expressed genes (log_2_FC, FDR <0.01, n = 4 biologically independent samples), between miR-211-hairpin-inhibitor treated rats and miR-211-mimic-treated rats. (**B**) Gene ontology (GO) analysis (ToppFun) for the DEGs that were identified to be different between the two treatment groups. (**C**) Mean gene expression of miR-211-hairpin-inhibitor-treated rats vs. miR-211-mimic-treated rats from the biological categories of ‘inflammation, immunity’, and ‘apoptosis, cell cycle’. Significant effect between groups is indicated by * 0.01 < *p* < 0.05, ** 0.001 < *p* < 0.01, *** 0.0001 < *p* < 0.001.

Given the opposing effects observed in feeding regulation with central miR-211-mimic and -inhibitor administration, we used RNA-seq to uncover the effect of miR-211 on the transcriptional landscape of the ARC. The number of genes differently expressed between miR-211-mimic and miR-211-inhibitor (FDR < 0.01) was 214. As the main role of miRs are to inhibit gene expression, it was not surprising that 90.6% (194/214) of DEGs were downregulated by miR-211-mimic (Figure 4A, Supplementary Table S3). GO analysis revealed that miR-211 is indeed involved in inflammatory suppression, including gene pathways associated with ‘cytokine response’, ‘regulation of the cell cycle’, and ‘apoptosis’ (Figure 4B). miR-211-downregulated DEGs related to the immune system included genes such as *Nfkbia* (t_6_ = 5.21, *p* < 0.01), *Cd44* (t_6_ =2.57, *p <* 0.05), *Bcl3* (t_6_ = 5.06, *p* < 0.01), and *Mt2a* (t_6_ = 4.35, *p* < 0.01). Further, we found downregulated genes related to cell cycle, including *Ccl19* (t_6_ = 3.32, *p* < 0.05), *Btg2* (t_6_ = 3.71, *p* < 0.001), *Gadd45b* (t_6_ = 6.02, *p* < 0.001), and *Bak1* (t_6_ = 4.57, *p* < 0.01) (Figure 4C). 

**Figure 5:**
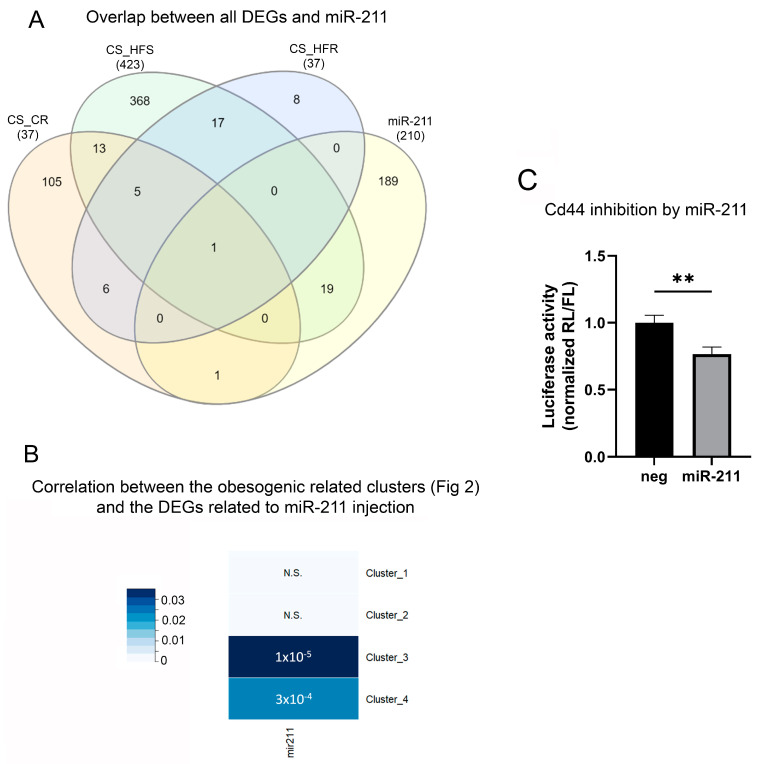
**miR-211 modulates the rescue of obesity induced-aberrantly expressed genes through exercise in the ARC.** (**A**) Venn diagram of differently expressed genes (FDR < 0.01) from the miR-211 administration experiment (n = 214 genes) and the different groups of the DIO/RW experiment. (**B**) Correlation analysis was performed between the DEGs linked to miR-211 administration (miR-211-mimic vs. miR-211-inhibitor) and the distinct clusters outlined in Figure 2 (CS vs. HFS). *p*-value (presented in numbers) and odds ratio (color scale) from Fisher’s exact were calculated by the GeneOverlap package and presented in the heat map. (**C**) Luciferase activity in HEK 293T cells cotransfected with psiCHECK-2 vector containing Cd44 3′ UTR and miR-211, or miR-negative control at 10 nmol. The experiment was performed independently three times, utilizing triplicate biological samples. The Renilla luciferase (RL) reporter signal was normalized to the firefly luciferase (FL) signal as an internal control. Data are presented as mean ± SEM. Significant effect between groups was determined using t test between the two groups. Significant effect between groups is indicated by ** 0.001 < *p* < 0.01, (non-significant N.S.).

Next, we assessed the overlap between the altered genes in DIO/exercise and genes that are differentially expressed between rats which were injected intracranially with miR-211 and miR-inhibitor. We found that 21 genes were mutually differentially expressed (Figure 5A). The majority of the overlapped genes were identified between the genes affected in the HFD-fed rats (Figure 5A) and among them were genes associated with ‘wound healing involved in inflammatory response’, ‘inflammatory response’, and ‘negative regulation of apoptotic signaling pathway’. Specifically, some of the genes involved include *Hmox1*, *Cd44*, and *Timp1*. Subsequently, we conducted a correlation analysis between the DEGs linked to miR-211 administration and the distinct clusters outlined in Figure 2. Our analysis unveiled a noteworthy correlation between the downregulated genes (miR-211-mimic vs. miR-211-inhibitor) and both cluster 3 and 4 (indicating an increase in HFD and rescue in HFR) as depicted in Figure 5B. These findings suggest a convergent beneficial effect between the impact of miR-211 on hypothalamic gene expression and voluntary running within the context of obesity (Figure 5B). 

As *Cd44* emerged in both the running-rescue cluster and was affected by the miR-211 injection, we validated its role as a target of miR-211 by confirming its binding to the 3′UTR seed site. To this end, we constructed a psi-check vector with the putative binding site of *Cd44* mRNA and transfected miR-211, or negative control. A dose of 10 nmol of miR-211 inhibited luciferase activity (*p* < 0.001), indicating that miR-211 indeed successfully binds to, and can inhibit, *Cd44* expression (Figure 5C).

## 3. Discussion

The benefits of living a healthy lifestyle by exercising and maintaining a balanced diet are vastly understood, but maintaining this lifestyle is often challenging for many individuals. The question then arises, is it beneficial to participate in some exercise, without making other lifestyle changes? 

In this work, we aimed to uncover what occurs on a physiological, genetic, and epigenetic level in the ARC when rats are given the opportunity to run voluntarily, with the background of obesity. We focused on microRNA modulation in this context because epigenetic modifications are dynamic and may be the driver for this reversible phenomenon. We found that along with reduced fat mass, upregulation of genes related to feeding behavior such as *Pomc* and *Cck* occurred. Exercise had a partial rescue effect on diet-induced hypothalamic inflammation, shown by patterns of transcriptional rebound of genes such as *Cd44, Rtl5, Rras*, and *Cd74*. Importantly, our data suggest that these alterations in gene expression within the ARC may be partially mediated by miR-211. This aligns with our transcriptomic focus and emphasizes the methodological approach employed in our study. This work highlights the role of miR-211 as an anti-inflammatory and neuroprotective agent in the hypothalamus, similarly to its previously published role in peripheral organs [32,33]. 

### 3.1. Voluntary Exercise Reduces Weight Gain and Body Fat in Diet-Induced Obese Rats

HFD-fed rats presented a higher BW compared to the chow-fed rats, even in the exercised group. Although exercise attenuated increase of BW in rats which maintained HF feeding, they did not reach baseline weight of CS. Voluntary exercise both in HFD and chow-fed rats reduced the amount of eating in the first 2–3 weeks of the experiment and reached sedentary levels in both treatments towards the end of the experiment. It is important to highlight that, initially, the rats fed a high-fat diet (HFD) consumed fewer grams of food compared to the chow-fed rats. However, when converting the food amount to calories, the HFD-fed rats consumed significantly more calories at the beginning of the experiment. These findings corroborate previous reports showing that exercising, while continuing consumption of energy-dense foods, resulted in less weight gain due to energy balance deficit from the increased movement [34]. The results presented in this study differ from those previously reported, which indicated sustained weight gain in female rats subjected to forced exercise [35]. These disparities may stem from the use of voluntary exercise in the current experiment, contrasting with the forced exercise employed previously. It is noteworthy that the high-fat diet fed rats in this study ran both less time and a shorter distance than the chow-fed rats, potentially influenced by the burden of overweight. As expected, the active rats had less fat tissue than the sedentary rats. Interestingly, the obese sedentary rats had more bone mineral deposited in the leg region, likely due to chronic weight-bearing on lower extremities due to excess weight [36].

It has been previously reported that male rats undergoing exercise show a reduction in food intake and a slower rate of weight gain. In contrast, female rats compensate for exercise by consuming more food, leading to a sustained rate of weight gain [35]. Since the phenotypic differences are clear, one would also expect gender differences in the molecular and epigenetic effects induced by voluntary exercise on the background of obesity. Recognizing the considerable effort involved in exploring these inquiries comprehensively across both genders, our project strategically focuses on females. This approach builds upon our prior experiments, revealing that females tend to demonstrate more pronounced responses in terms of weight gain or loss [15]. The question of the molecular/epigenetic correlates of exercise in males and sex differences should be addressed separately. 

The chow-running group exemplifies an optimal and healthiest lifestyle among the studied rats. These rodents, given the freedom to engage in voluntary running activities, display an inclination towards utilizing the exercise wheel. Intriguingly, their tendency for running intensifies over time, emphasizing the intrinsic appeal of physical activity for these animals. Notably, their dedication to running is so pronounced that it translates into reduced time spent on eating, resulting in a lower overall food consumption compared to their sedentary counterparts. In parallel, the transcriptional signature of these rats differs from the CS rats that are basically non-active. The gene expression pattern of the clusters that were unbiasedly created demonstrate that there is an opposite effect when comparing the HFS and the CS in respect to the CR rats. Drawing from both previous publications [7,8], we can assume that voluntary running optimizes brain activity, a process disrupted by the low levels of inflammation in the high-fat sedentary rats. However, the partial rescue effect of running in the obesogenic background brings the hypothalamic gene expression pattern of the HFR closer to the levels of the CR. 

### 3.2. Voluntary Physical Activity, on the Background of HFD-DIO Affects the Expression of Functional Gene Families in the ARC

The primary objective of this project is to underscore the benefits of voluntary exercise on molecular and epigenetic processes within the hypothalamus. We theorized that by optimizing behavioral outcomes, we will be able to identify significant molecular changes. When comparing DIO animals to their chow-fed counterparts, a discernible transcriptional signature emerges. This signature is predominantly associated with altered feeding regulation, elevated levels of inflammation, an upregulated innate immune response, and observable impairments in brain development and synaptic modulation (Figure 2B,C). In alignment with these findings, it has been well established that obesity is linked to chronic low-grade inflammation, partly induced by alterations in muscle tissue [37] and adipose tissue, facilitated by macrophage activation [38]. These complementary results further underscore the interplay between molecular processes and the systemic effects of obesity.

We hypothesized that exercise might have beneficial outcomes on the brain, even when the animals are maintained on HFD. Of particular interest, we found that the *Rescue up* clusters containing genes that are upregulated in HFD condition but are reduced due to exercise were highly enriched for genes responding to ‘feeding regulation’, ‘T-cell differentiation’, ‘wound healing’, ‘glial cell development’, and ‘cytokine response’. Genes in these cluster include *Pomc*, *Cck*, *Cd44*, *Cd74*, *Mt2a*, *Cxcl16*, and *HSPB*. The identification of genes like *Pomc*, which exhibit altered expression patterns in obesity, as demonstrated in our research and by others [16,17,18,19,20], enhances our confidence that the list of rescued genes identified here is indeed relevant. Furthermore, systemic inflammation is a hallmark of obesity, and here we show that pro-inflammatory transcripts are at least partially downregulated in active obese rats (HFR). Pro-inflammatory glucocorticoid signaling is triggered by the binding of macrophage migration inhibitory factor to the Cd74/Cd44 receptor [39]. Even preceding substantial weight gain, a few days of HFD-consumption triggers inflammatory changes such as astrocytes and microglia recruitment and markers of neuronal injury in the hypothalamus [40]. Activation of inflammatory pathways in the hypothalamus of lean, chow-fed rodents results in obesity, showing that hypothalamic inflammation is not only a pathological outcome of obesity, but an active player in the development and progression of obesity. Our findings support these reports, showing that these genes are involved in obesity-induced inflammation. Further, our novel findings indicate that they can be rescued with moderate exercise in the hypothalamic ARC. 

In addition to genes involved in inflammation regulation, we found a rescue pattern of genes related to apoptotic processes and cellular proliferation. Recently there has been evidence of continuous cellular remodeling in the different hypothalamic regions, including the ARC. HFD led to increased apoptosis [3,41] of newly generated neurons and decreased generation of new neurons, resulting in fewer synapses of POMC and AgRP neurons [1]. The enrichment pattern suggests that physical activity has distinct beneficial outcomes on neural functions, and further that the clusters outline novel targets that may be advantageous results of voluntary exercise in obese rats.

### 3.3. miR-211 Modulates Feeding Behavior and Is Involved in Inflammation Processes in the ARC

MiRs have previously been implicated in the regulation of both peripheral metabolic pathways and central mechanisms. Furthermore, the benefits of exercise have been correlated with the levels of miR expression [42]. The role of miRs in obesity-associated disorders and the impact of exercise training on the heart, vascular tissue, and adipose tissue have been extensively reviewed [43]. Additionally, lifestyle factors, including diet and exercise, have been shown to influence the incidence, recurrence, and mortality rates related to cancer among breast cancer (BC) survivors, with miR regulation playing a key role [44,45]. In the satiety centers, in the hypothalamus, microRNA-33 has been demonstrated to controls hunger signaling in hypothalamic AgRP neurons [46]. Despite these findings, there are likely many miRs involved in a complex interplay that regulates both obesity and the beneficial effects of exercise on health issues associated with obesogenic traits [42].

Identifying upregulation of inflammatory-related genes such as Cd44 in response to a high-fat diet (HFD) is noteworthy, and their subsequent rescue through exercise raises questions about the modifying agent. In parallel, miR-211 is also upregulated in the presence of HFD, suggesting a potential regulatory role. While we identified an overlap between several genes predicted to be controlled by miR-211 (Figure 3A) and the downregulated genes in the high-fat sedentary (HFS) group (Figure 2C), surprisingly, it seems that several other genes remain upregulated. One plausible explanation lies in the correlation between obesity and low-grade inflammation in the hypothalamus. It is conceivable that miR-211 plays a role in modulating inflammatory processes, acting as a suppressor to maintain homeostasis. However, the elevation in the expression pattern of *miR-211* in the chronic steady state of DIO is probably not enough to block the natural occurring elevation in the expression of these aforementioned inflammatory genes. Without the upregulation of miR-211, there might be a significant surge in inflammation. Thus, it seems that miR-211 may serve as a key regulatory element, preventing an escalation of inflammatory responses in the context of obesity.

To establish the role of miR-211 in the ARC in the context of feeding control, exogenous administration of miR-211 to the ARC was performed. Indeed, increased food intake was observed 4 h and 6 h after miR-211-inhibitor injection. In addition, some inflammatory related genes, i.e., *Btg2* and *Cd44*, were downregulated in the miR-211-mimic injected rats, which strengthens the possibility that this regulation mechanism is involved in the weight control phenotype (Figure 5A). It should be emphasized that this experiment addressed only eating motivation, without directly addressing the HFD or the voluntary exercise effect. 

To identify new candidates that may influence hunger/satiety in the arcuate nucleus (ARC), we generated a list of 21 candidate genes modulated by both miR-211 administration and DIO. Among those genes, we identified clusters related to inflammation and immunity, including cytokines and wounding, as well as cell cycle regulation. An example of such a gene is *Cd44*, which is both upregulated in obesity, rescued by voluntary exercise, and downregulated by injection of miR-211. Indeed, these findings corroborate the hypothesis that the expression of miR-211 is amongst the mechanism that controls inflammation in the ARC. To verify that Cd44 indeed is a target of miR-211, in vitro luciferase assay confirmed the binding of miR-211 to *Cd44*. 

In theory, an obesogenic environment should trigger an upregulation of anorexic neuropeptides in the ARC. However, our findings reveal an unexpected upregulation of *Pomc* in DIO rats. Several factors may account for this phenomenon. Firstly, prolonged exposure to a high-fat diet could induce stress, influencing the expression of *Pomc*. Additionally, our prior research has indicated malprogramming in the ARC signal transduction pathway during obesity, leading to the disruption of *Sp1* binding to the hypermethylated Pomc promoter and, consequently, non-elevated levels of *Pomc* in DIO rats [32]. Interestingly, we observe a similar paradoxical upregulation of *Pomc* in rats subjected to a high-fat diet. However, voluntary running markedly mitigated this effect.

### 3.4. Voluntary vs. Obligatory Exercise in DIO Rats

Utilizing either obligatory or voluntary exercise protocols within the context of an obese phenotype presents both merits and drawbacks. Implementing obligatory exercise would ensure uniformity in exercise parameters between obese and control rats, holding promise for facilitating direct comparisons and elucidating the effects of exercise intervention on both groups. However, imposing obligatory exercise on obese rats introduces several potential challenges. Notably, it may exacerbate the burden on these animals compared to their control counterparts, potentially leading to heightened stress levels among the obese cohort. Stress, particularly in the context of obesity, can evoke physiological responses that may confound experimental outcomes and interpretation. Moreover, the increased effort required for exercise engagement in obese rats could perturb their food consumption patterns, thereby complicating the assessment of metabolic responses and overall health outcomes.

Previous studies have demonstrated that forced and voluntary exercise differentially affect brain and behavior. In a project in which both voluntary and forced exercise groups covered the same distance, voluntary exercisers ran at higher speeds and for less total time than forced exercisers. When compared with sedentary controls, forced but not voluntary exercise was found to increase anxiety-like behaviors in the open field [47]. Furthermore, on the molecular level, voluntary exercise protects against stress-induced decreases in brain-derived neurotrophic factor protein expression [48]. Since one of the important goals of this project was to demonstrate the benefits of participating in some exercise without making other lifestyle changes and to elucidate the repertoire of genes in the ARC that are rescued by exercise on an obese background, we chose to use the voluntary exercise model in this project. However, it should be noted that studying forced exercise would contribute to the knowledge acquired here.

## 4. Limitations, Future Directions, and Conclusions

The findings presented here expand the repertoire of genes altered by obesity and rescued by voluntary exercise, emphasizing the role of inflammation in the hypothalamus as a key factor in the obesogenic phenotype. Additionally, they highlight miR-211 as an important target for intervention. miRs are increasingly recognized as critical regulators of host homeostasis and the innate immune response, including the maintenance of immune equilibrium [42]. Understanding the role of miRs in correcting dysregulated gene expression in the hypothalamus is essential for combating obesity, which has become a global epidemic.

In recent years, significant progress has been made in designing and strategizing the delivery of miR mimics or antagomiRs [49]. These advanced approaches hold great promise for developing therapeutics aimed at targeting the metabolic and inflammatory disruptions associated with obesity. By modulating miR-211, it might be possible to influence key pathways involved in appetite regulation and inflammatory responses, potentially offering new avenues for effective treatment of obesity and related metabolic disorders.

Further research into miRs’ mechanisms of action and their interactions within the hypothalamus could pave the way for innovative therapies that not only address the symptoms of obesity but also its underlying causes. This could lead to more sustainable and effective interventions, improving health outcomes for individuals affected by obesity and its associated complications.

A notable limitation of this research is its inability to account for cell composition within the target tissue. The arcuate nucleus is a heterogeneous structure composed of various cell types, with only about half of its cells being neurons. Among these neurons, satiety/hunger neurons represent only a small fraction. Consequently, without a method to control for cell composition, it is challenging to determine whether observed differences in gene expression are due to specific changes within certain cell types or shifts in the overall composition of different cell types. To address this limitation, future experiments should include gene expression analysis with identified cell types, potentially through cell sorting techniques. This approach would allow for a more precise understanding of how specific cell populations contribute to the observed gene expression changes. 

In conclusion, our study provides evidence that physical activity, even without significant weight loss or dietary changes, can profoundly benefit the health of individuals with obesity. Specifically, we have delineated the repertoire of genes that undergo reversal in the arcuate nucleus of obese rats following voluntary physical activity. It is noteworthy that among these genes are those associated with low-grade inflammation. Furthermore, we elucidate the potential role of miR-211 in regulating these genes, both as a primary target and as secondary effects of the pathways influenced by miR-211 targets. These findings are particularly encouraging, as they suggest that even modest increases in physical activity can yield significant positive impacts on obesity-related health outcomes. Our research underscores the crucial importance of regular exercise as a means of enhancing health status and fostering overall well-being in the population.

## 5. Material and Methods 

### 5.1. Animals

All experimental procedures were approved by the Bar-Ilan University Animals Care and Use Committee and were performed in accordance with the American Psychological Association and Society for Neuroscience guidelines. All efforts were made to minimize suffering and the number of rats used.

Female Wistar rats were bred at Envigo RMS (Jerusalem, Israel) and brought to Bar-Ilan University’s rodent facility at standard weaning age (post-natal day 21 (PND)). Rats were fed either standard chow (2018SCF; Teklad Global 6% Fat Rodent Diet; Harlan, Madison, WI, USA) or a 60% high-fat diet (D12492; Research Diets, Inc., New Brunswick, NJ, USA), depending on experimental group. Rats were given free access to water and their designated diet ad libitum throughout the entire study. Room temperature was maintained at 22 ± 2 °C, with a standard 12-hour lights on/off schedule. Each home cage was equipped with standard rodent bedding and plastic tube for enrichment. 

### 5.2. Voluntary Wheel Running after Diet-Induced Obesity

The experiment was conducted twice. In Phase I of each experiment, rats were raised from PND 21-90 on either chow (total n = 12 in each experiment) or HFD (total n = 12 in each experiment) in pairs, in standard cages with plastic enrichment tubes. In Phase II, rats were housed individually in PhenoMaster cages (TSE Systems Inc., Bad Homberg, Germany), with exposure to a running wheel, or a sedentary environment. Rats were assigned into one of the four treatment groups with care to equalize body weight between diet groups: Chow-Sedentary (CS), Chow running (CR), High-fat Sedentary (HFS), and High-fat Running (HFR) (n = 6 per experimental group in each experiment, total n = 12 per group). The experimental computerized activity cages were equipped with stainless-steel running wheels with a diameter of 25cm. The non-running cages did not have a wheel, but a plastic tube was provided to supplement enrichment. The PhenoMaster system collected feeding and running data duration over the 4-week experiment. Rats were weighed on a weekly basis. At the end of Phase II, rats were sacrificed. 

Rats underwent a training protocol one week prior to Phase II. During training, they were transferred in pairs into experimental PhenoMaster cages with novel water bottles and food hoppers overnight for 4 consecutive nights to learn how to feed and drink from the new equipment. During the light hours, rats were housed in their home cages and continued to eat and drink as usual. This protocol ensured that normal amounts of food and water were consumed in the days leading up to the transfer into individual experimental cages. 

### 5.3. Intracerebroventricular Administration miR-211

#### 5.3.1. Surgery and Cannula Implantation

Female Wistar rats were bred at Envigo RMS (Jerusalem, Israel) and brought to the Bar-Ilan University’s rodent facility at PND 60 (130–140 g, n = 30). They were assigned randomly and housed in groups of 3 during the acclimation and handling period for two weeks. Rats were given free access to water and ad libitum chow throughout the study. Room temperature was maintained at 22 ± 2 °C, with a standard 12 h lights on/off schedule. Experiments were conducted during the dark hours. 

For cannula implantation, rats were anesthetized intraperitoneally (i.p.) with ketamine hydrochloride (100 mg/kg) and xylazine (10 mg/kg) (Sigma-Aldrich-Merck, Jerusalem, Israel). A 22-gauge guide cannula was implanted with the aid of a stereotactic device (David Kopf Instruments, Los Angeles, CA, USA) into the 3rd ventricle (coordinates from Bregma [50]): AP: −2.12 mm; L: 0 mm; DV: 7.5 mm) and sealed with a cannula dummy (Plastics One, Roanoke, VA, USA), then secured to the skull with screws and dental acrylic cement. Gentamicin was applied to the exposed tissue to minimize infection. A cocktail of Baytril (0.4 mg/kg) and Carprofen (0.1 mg/kg) (Sigma-Aldrich-Merck, Jerusalem, Israel) were administered subcutaneously (S.C.) after surgery, and for two days post-op. The rats were given 10–14 days to recover before undergoing intracranial ventricular infusion. During this time, the rats were given free access to chow and water.

#### 5.3.2. Feeding Assessment

Rats were fasted overnight before assessing food intake, in order to ensure a uniform hunger level between all rats [51]. Chow was replaced at the onset of the dark hours (t = 0 h), and weighed manually at time points 2 h, 4 h, and 6 h after onset of dark phase. Rats were assigned into one of the three treatment groups with care to equalize body weight between groups (n = 10 per group). On the day of the experiment, at t = 0, intracranial ventricular infusion the various miR compounds was administered (see below). The internal cannula remained in place for 5 min after the infusion. Rats were returned to their cages with access to chow. Chow was weighed manually at time points 2 h, 4 h, and 6 h after administration. At hour 6, rats were sacrificed. The experimenter was blind to the treatment when measuring the food intake. The placement of the guide cannula was verified by visual inspection when frozen brains were sliced using the cryostat. 

#### 5.3.3. Pharmacological Compounds

MiRIDIAN hsa-miR-211 mimic, MiRIDIAN hsa-miR-211-hairpin inhibitor, and MiRIDIAN mimic-negative control (Horizon, Cambridge, UK) were dissolved in BrainFectIN (Oz Biosciences, Marseille, France) to a concentration of 1 μg/μL according to manufacturer’s instructions. Each compound was prepared freshly before administration. The solution (total volume 2.5 μL) was infused for 5 min. 

### 5.4. Tissue Collection

#### 5.4.1. Sacrifice

At the end of each experiment, rats were sacrificed via rapid decapitation after brief CO_2_ exposure. Brains were removed and immediately frozen on dry ice and stored at −80 °C.

#### 5.4.2. Neural Tissue Extraction

Coronal brain sections of the hypothalamus were sliced using a cryostat (−2.3 to −4.5 mm Bregma, using Paxison and Watson coordinates) and the ARC was extracted with a 1 mm disposable Miltex biopsy punch plunger (Bar Naor Ltd., Petah Tikva, Israel). Punches from each hemisphere were immersed in RNA Save (Biological Industries, Kibbutz Beit-Haemek, Israel) for downstream RNA extraction. 

### 5.5. DEXA

The body composition of the subcranial corpses was measured using the Lunar PixiMus dual-energy X-ray absorptiometry (DEXA) (software version 1.42.006.010; Lunar Corp, Madison, WI, USA). Calibration of the instrument was performed according to the manufacturer’s instructions. Because of the large size of the rodents, four anatomical regions of the thawed corpses were scanned separately: chest, abdomen, legs, and tail. The size and area of region of interest (ROI) for each anatomical structure was standardized for each subject. Analysis of the scans yielded measurements of bone mineral density (BMD) in mg/cm² and % fat tissue. DEXA analysis was conducted in 4–6 rats per treatment group. 

### 5.6. Total RNA Extraction

RNA was isolated using TriReagent (Molecular Research Center, Cincinnati, OH, USA) from samples from DIO/RW experiment, and RNA/DNA Purification Micro Kit (Cat. 50300) (Norgen BioTek Corporation, Thorold, ON, Canada) from samples from miR-injection experiment, each according to the manufacturer’s instructions. 

#### 5.6.1. Micro-RNA Quantification

Mature miRNA levels were quantified using hsa-miR-211 TaqMan MicroRNA assays (Applied Biosystems, Waltham, MA, USA) according to the manufacturer’s instructions. Briefly, 10 ng of total RNA was reverse-transcribed using TaqMan MicroRNA Reverse Transcription Kit and miRNA-specific RT-primer, followed by real-time PCR with TaqMan probes (Applied Biosystems, Waltham, MA, USA). Within each sample, relative miRNA expression was normalized to that of Hprt mRNA.

#### 5.6.2. RNA-Seq

RNA-Seq protocol for the voluntary running experiment and the miR-211 injection were performed similarly. RNA-seq libraries were prepared at the Crown Genomics institute of the Nancy and Stephen Grand Israel National Center for Personalized Medicine, Weizmann Institute of Science, Rehovot, Israel. A bulk adaptation of the MARS-Seq protocol [52,53] was used to generate RNA-Seq libraries for expression profiling of all 4 voluntary experiment groups (n = 3 CS and CR, n = 4 HFS, and HFR) and the 2 miR-211 experimental groups (n = 4 for both treatment groups i.e., miR-211-mimic and miR-211-hairpin-inhibitor). Briefly, (30 ng of input) RNA from each sample was barcoded during reverse transcription and pooled. Following Agencourt Ampure XP beads cleanup (Beckman Coulter, Brea, CA, USA), the pooled samples underwent second strand synthesis and were linearly amplified by T7 transcription. The resulting RNA was fragmented and converted into a sequencing-ready library by tagging the samples with Illumina sequences during ligation, RT, and PCR. Libraries were quantified by Qubit and TapeStation, as previously described [54,55]. Sequencing was done on a Nextseq 75 cycles high output kit (Illumina, San Diego, CA, USA). All RNA-Seq data were deposited to the Gene Expression Omnibus database under accession number GSE232510.

#### 5.6.3. RNA-Seq Analysis

Trimming of Illumina adapters was performed with Cutadapt (V3.4). The raw fastq data were aligned to the Rnor6 reference genome using STAR (v 2.7.9a) with an 86% alignment rate, resulting in an average of 11,371,935 mapped reads per sample. 

The SAMtools collection [54] was used to sort, remove PCR duplicates (rmdup), index BAM files (index), and calculate library statistics (unique mapped aligned—9,313,424; feature count—2,251,492). To estimate transcript abundances, the mapped reads were processed by HTseq (v 0.13.5), with the following parameters: ‘htseq-count -s no --mode = union’. Finally, ‘read count’ matrix was assembled from all the tested RW groups and pair-wise differential gene expression analysis was performed using DESeq2. To elucidate genes alterations, we assessed the fold change expression of DEGs (CS vs. HFS, n = 430), across all groups (i.e., CS, CR, HFS, HFR), with 61 relative to the CS group and unbiasedly clustered (k-mean) them with R packages; Circlize (0.4.15), ComplexHeatmap (2.18), gridExtra (2.3), and viridis (0.6.4). Respective heatmaps were generated by custom R scripts and the Complexheatmap R package. HOMER package [55] was used for Gene Ontology (GO) analysis and Enrichr was used for TF motif enrichment analysis [56,57,58].

### 5.7. Luciferase Assay

Luciferase assay protocol was performed as previously described [59]. To construct the luciferase reporter vector, a Cd44 3′-UTR fragment containing potential miR-211-5p-binding site (AAAGGGA) was PCR-amplified using primers (5′→3′): F-GGGAGTAGGAGAGCTTCTGTC and R-ATGCCTCCTCATGCCAATCT. The PCR product was inserted into PGEM-T Easy vector (Promega, Madison, WI, USA). Then, the insert was cut from the PGEM-T vector by NotI restriction enzyme (New England Biolabs, Ipswich, MA, USA) and cloned into plasmid psiCHECK-2 (Promega, Madison, WI, USA), which had been previously opened by NotI and dephosphorylated by calf intestinal alkaline phosphatase (CIAP; both from New England Biolabs, Ipswich, MA, USA). The presence of the Cd44 3′-UTR fragment and its orientation in the psiCHECK-2 plasmid were verified by sequencing analysis (Hylabs, Rehovot, Israel).

PsiCHECK-2 vector (200 ng) with confirmed Cd44 3′-UTR fragment was cotransfected with 10 pmol miRIDIAN rno-miR-211-5p mimic or 10 pmol miRIDIAN miR-negative-control (both from Dharmacon, Lafayette, CO, USA) into HEK-293T cells in 24-well plates using lipofectamine 2000 (Invitrogen, Waltham, MA, USA). Twenty-four hours post-transfection, cells were washed with phosphate-buffered saline (PBS) and lysed in Passive Lysis Buffer (Promega, Madison, WI, USA). For each transfection, firefly and Renilla luciferase activities were determined using the Dual-Luciferase Reporter Assay System (Promega, Madison, WI, USA) according to the manufacturer’s instructions and read in a VictorLight 1420 luminescence counter (PerkinElmer, Waltham, MA, USA).

### 5.8. Statistical Analysis

Data were analyzed using GraphPad Prism 8 software (San Diego, CA, USA). All data were examined for normality by goodness of fit test and by Bartlett’s test for variance equality. As the distribution was normal, the parameters were not transformed. There was no sample size calculation; sample size was determined on the basis of previous studies [14,60]. T tests for independent samples were used to compare between groups in experiments with two treatments and one-way ANOVA for multiple comparisons in experiments with three treatments. Two-way repeated measures ANOVA was used to analyze body weight between treatment groups over time. Tukey’s multiple comparison test was used to reveal treatment differences. Data are presented as means ± standard error of the mean (SEM). In the text, statistical values (t and F) and their significance (P) are reported, as well as post hoc multiple comparisons, where appropriate. The symbols in the figures indicate significance between groups, either by t test or post hoc (# *p* < 0.1, * *p* < 0.05, ** *p* < 0.01, *** *p* < 0.001).

## Data Availability

The data that support the findings of this study are openly available in NCBI GEO, with accession number GSE232510. The rest of the data are contained within the article and Supplementary Materials.

## Supplementary Materials

The following supporting information can be downloaded at: https://www.mdpi.com/article/10.3390/ijms25137188/s1.
