# Supplementary figures and images for: Exercise Rescues Obesogenic-Related Genes in the Female Hypothalamic Arcuate Nucleus: A Potential Role of miR-211 Modulation
Source: Int J Mol Sci. 2024 Jun 29;25(13):7188. doi: 10.3390/ijms25137188 (PMC11241292; doi:10.3390/ijms25137188)

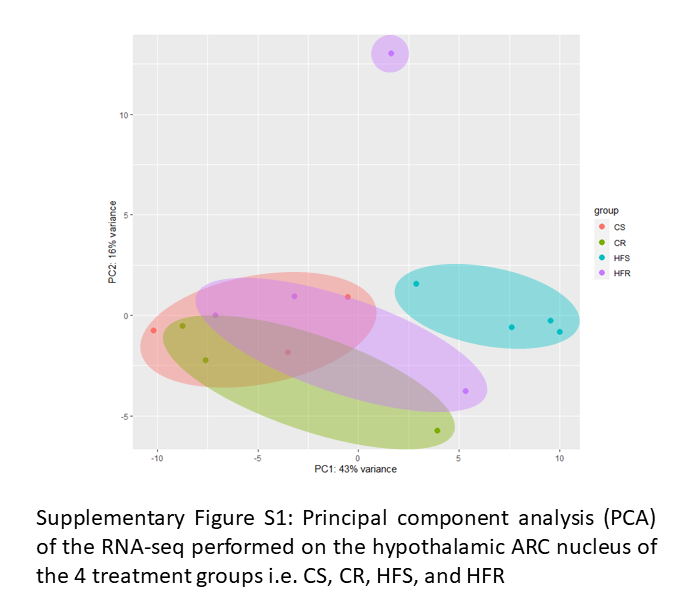

Supplement: Supplementary file 1 [file ijms-25-07188-s001.zip › supplementry Figure S1.tif]

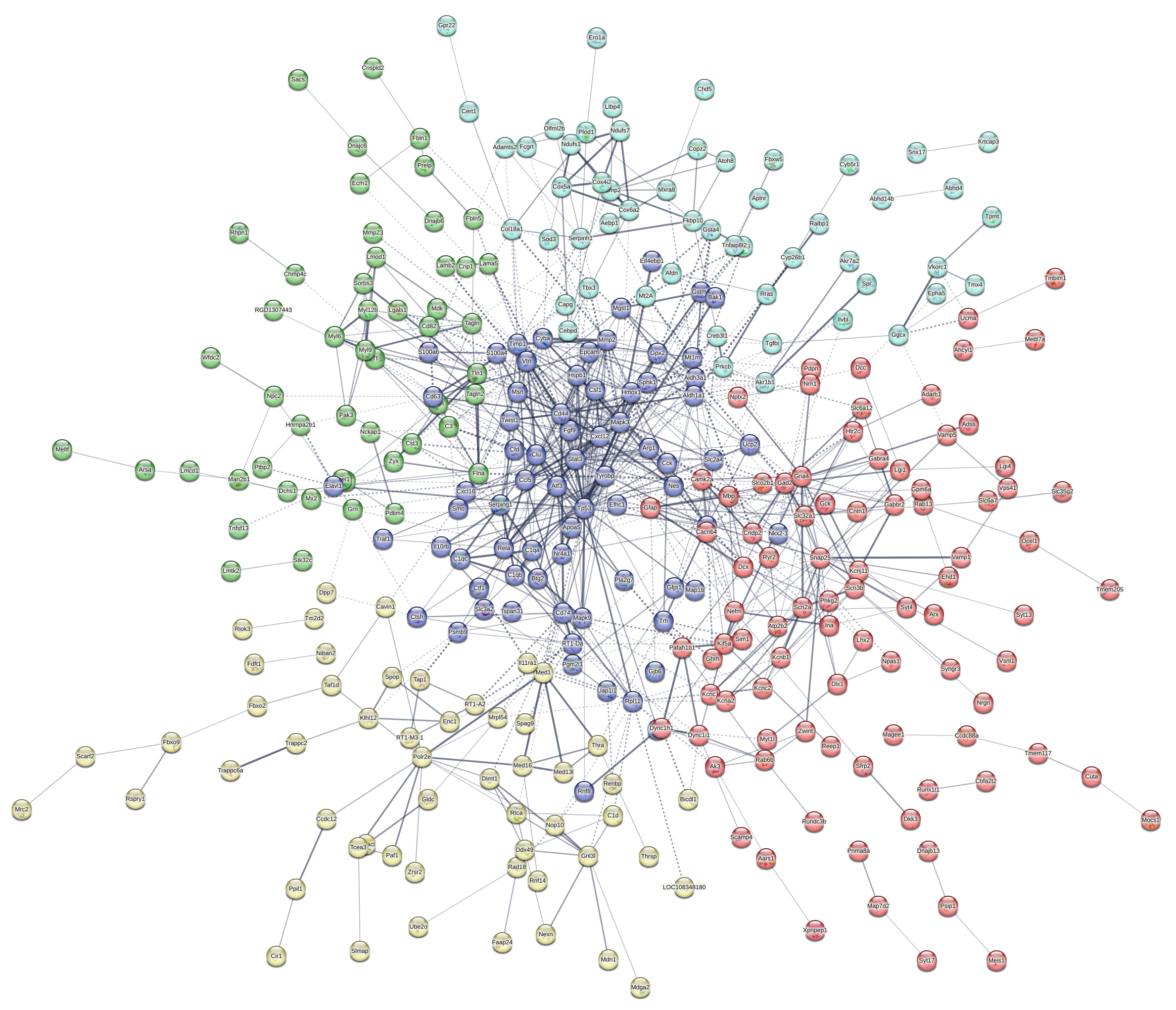

Supplement: Supplementary file 1 [file ijms-25-07188-s001.zip › Supplementry Figure S2.tif]
